# Validation of a dry‐slide immunoassay for progesterone analysis in canine plasma in a clinical setting

**DOI:** 10.1111/vcp.13140

**Published:** 2022-07-19

**Authors:** Sarah Østergård Jensen, Josefine Öberg, Helene Alm, Bodil S. Holst

**Affiliations:** ^1^ AniCura Small Animal Referral Hospital Bagarmossen Stockholm Sweden; ^2^ The Swedish University of Agricultural Sciences Uppsala Sweden

**Keywords:** laboratory analysis, method comparison, ovulation, reproduction

## Abstract

**Background:**

The identification of canine ovulation is critical for successful breeding. Progesterone measurements are useful for identifying ovulation. Progesterone assays are also quantitative and easily accessed, making them valuable in veterinary practice.

**Objectives:**

We aimed to validate a dry‐slide immunoassay (DSI) for use in dogs, including a method comparison with the chemiluminescence assay (CLIA) and mass spectrometry.

**Methods:**

Twenty‐nine bitches were prospectively recruited. Accuracy, precision, interference, and stability were evaluated. Method comparison between DSI and CLIA and mass spectrometry was conducted, and bias was calculated.

**Results:**

Repeatability was 8.0%–10.8%, and within‐laboratory imprecision was 8.8%–11.1% for four concentration levels. Recovery under dilution was 61%–100%, and the method was linear to a concentration of ~50 nmol/L. Recovery after the addition of a high progesterone sample was 76%–83%. Minor changes were seen in one hemolytic and two lipemic samples. Storage at room temperature for 12–24 hours resulted in concentrations that were 57%–96% of the initial concentrations. For samples frozen at −80°C, the concentrations were reduced 17%–27%. There was a significant difference between results from the DSI and CLIA, and a proportional bias was seen when DSI was compared with mass spectrometry, where CLIA correlated better than DSI.

**Conclusions:**

Precision and accuracy were acceptable. A proportional bias was seen between DSI and CLIA. A small amount of interference was seen with hemolysis and lipemia. Progesterone concentrations were decreased in samples stored at room temperature and −80°C. The results support the use of the DSI for ovulation timing but not for artificial insemination with frozen semen since progesterone concentrations might exceed the assay's linearity and precision limits.

## 1 INTRODUCTION

The canine estrous cycle is divided into four phases: proestrus, estrus, diestrus, and anestrus.^1^ In proestrus, serum estradiol concentrations rise, peaking between 0.5 and 3 days before the end of proestrus, after which time it starts to decline.^1^ The elevated concentration of estradiol during proestrus initiates the release of luteinizing hormone (LH) that precedes ovulation.^1^, ^2^, ^3^ During the estrus, estradiol concentrations continue to decrease.^1^, ^2^, ^3^ The serum progesterone concentration increases quickly at the time of the LH surge,^1^, ^3^, ^4^, ^5^ continues to increase during estrus, and peaks during the diestrus, at cycle day 20–35.^1^ The end of diestrus is marked by low serum progesterone concentrations.^1^ The canine estrus cycle differs from most domesticated animals in that it has both a long estrus and anestrus phase.^6^ The estrus phase can range from 6 to 11 days.^1^ The combination of a transition from proestrus to estrus that can be difficult to determine, and the fact that the proestrus‐estrus phase can range from 10 to 35 days in dogs, results in great variability for the fertile period.^1^ The long estrus phase results in difficulty in determining when ovulation takes place, which is essential for high pregnancy rates. The long anestrus phase results in few chances for mating per year for most dogs.^1^, ^6^, ^7^ Adding to this, the possibly long distances traveled for natural mating and the increasing use of artificial insemination (AI) with chilled and frozen semen necessitate accurate identification of ovulation time for the best chances of fertilization.^1^, ^5^, ^7^, ^8^, ^9^, ^10^

Different methods have been used to determine the optimal time for mating such as studies of behavioral changes, clinical signs, ultrasound of the ovaries, and vaginal cytology.^5^, ^8^, ^9^, ^10^, ^11^, ^12^, ^13^, ^14^ Apart from ultrasound, those methods do not predict the LH surge and subsequent ovulation. It is, therefore, advisable to complement these analyses with reproductive hormone measurements, primarily progesterone measurements.^1^, ^4^, ^12^, ^13^

Ovulation occurs approximately 48–60 hours after the LH surge,^1^ but since daily blood sampling is required to avoid missing the LH surge, measuring LH to determine ovulation is not useful in clinical practice.^1^, ^5^, ^13^ The LH surge can take from 24 to 60 hours, and there is great individual variation.^4^ Ovulation in the dog takes at least 24 hours, which means that there can be up to 24 hours between the first and last ovulations and concurrent luteinization that leads to a rise in progesterone.^14^ Progesterone analysis plays a significant role in determining when ovulation takes place.^5^, ^7^, ^8^, ^9^, ^13^, ^14^ The changes in progesterone concentrations around ovulation happen quickly, making analytical precision and accuracy important.^1^, ^4^, ^13^, ^15^

Radioimmunoassays (RIAs) have been traditionally used to measure progesterone concentrations. These assays are mainly found in commercial laboratories and are considered the gold standard in method comparison.^13^, ^16^ Mass spectrometry has been used as the gold standard for quantifying steroid hormones, and it has been shown to correlate well with RIA when used for progesterone analysis.^17^ Using mass spectrometry in clinical practice is limited since it is not readily available, and samples usually need to be shipped.^17^, ^18^, ^19^ Quantitative assays for the measurement of progesterone in serum or plasma that do not use radioactivity have been developed.^2^, ^4^, ^10^, ^11^, ^15^, ^16^, ^20^, ^21^, ^22^

Differences between instruments and methods used for progesterone analysis have been reported, and concentration around ovulation have been found to be between 8.8 and 20.0 nmoL/L, depending on the method used^1^, ^4^, ^11^, ^13^, ^15^, ^22^ Individual validation for each assay is necessary before clinical use.^15^

With the hormonal changes described above, and the need for fast clinical decision‐making, reliable in‐house assays have a significant value for both veterinarians and breeders. Instruments used for this type of analysis should be accessible, easy, and safe to use for operators. They should be precise, accurate, and ideally correlate well with other assays since serial analysis at different locations can be needed.

Progesterone analysis on the IDEXX Catalyst Dx (IDEXX Nordics, Solna, Sweden) uses an immunoassay technique for the quantitative measurement of progesterone with a reportable range of 0.6–63.6 nmol/L.^18^ The instrument is an in‐house analyzer available in hospitals as well as small clinics. A recent study has evaluated precision and method comparison with the chemiluminescence assay (CLIA), but an objective validation study, including dilution, recovery, interference testing, stability, and method comparison with mass spectrometry has not yet been provided.^22^

The Immulite 2000XPi (Siemens Healthcare AB, Solna, Sweden) is a validated instrument using chemiluminescence for progesterone analysis in dogs.^2^ Several studies using Immulite 2000 for progesterone analysis have been published.^2^, ^16^, ^21^ It has a reportable range of 0.64–127.0 nmol/L and is used in clinics and hospitals around the globe. It is an instrument that demands maintenance and trained staff, which means that it is not eligible for all clinics.

The objectives of the study were to validate the Catalyst Dx progesterone dry‐slide immunoassay in a clinical setting. We also evaluated agreement between DSI and CLIA and agreement between both assays and mass spectrometry.

## 2 MATERIALS AND METHODS

### 2.1 Study population

This prospective study used samples from patients visiting the reproduction department at the AniCura Small Animal Referral Hospital Bagarmossen, Sweden, between May 15, 2019, and February 10, 2022. The reproduction department employs a specialized veterinarian and nurse. Ethical permission was granted by the Swedish Board of Agriculture, with ID number: 5.8.18–17 395. The Inclusion criteria were intact, nonpregnant female dogs that were healthy according to information from owners and on clinical examination. Owners were informed about the study, and written consent was obtained prior to blood sampling. Exclusion criteria were dogs where the owner declined participation, dogs with a history of sampling difficulties, or difficulties with sampling at the visit.

### 2.2 Blood collection and handling

Sampling was performed using a standard aseptic technique. Blood was drawn from the cephalic or jugular vein using a BD vacutainer Safety‐Lok blood collection set (23G x ¾” x 7″; Becton Dickinson AB, Stockholm, Sweden), and collected in 2 ml BD vacutainer tubes; one clot‐activator serum tube (CAT) and one lithium‐heparin tube. Serum tubes were used for analysis with the chemiluminescence assay (CLIA) (Siemens Healthcare AB, Solna, Sweden, and lithium‐heparin tubes were used for analysis using a dry‐slide immunoassay (DSI) (IDEXX Nordics, Solna, Sweden) and mass spectrometry (UPSFC–MS/MS, ACQUITY UPC^2^; Waters Corporation, Milford, MA, USA). Serum tubes were allowed to clot for 60–120 minutes prior to centrifugation.^23^, ^24^ Lithium‐heparin tubes were centrifuged within 30 minutes from blood collection, as recommended by the DSI manufacturer.^18^ Both lithium‐heparin and serum tubes were stored at 18–25°C until centrifugation and centrifuged at 1530 *g* for 5 minutes on a Hettich Universal 32 centrifuge (Hettich labinstrument AB, Stockholm, Sverige). Plasma and serum were separated and aliquoted into Sarstedt 0.5, 1.0‐, or 2.5‐mL plastic tubes (Sarstedt AG & Co. KG, Nürmbrecht, Germany) and analyzed directly thereafter with either DSI or CLIA. Aliquots were stored at a −80°C freezer directly after analysis.

### 2.3 Study design

The validation study was set up in concordance with the “ASVCP quality assurance guidelines: control of general analytical factors in veterinary laboratories”.^25^ For the precision study, the Clinical and Laboratory Standards Institute (CLSI) document EP15‐A3^26^ was used, and for bias and interference evaluations, the CLSI document EP7‐A2^27^ and “method comparison in the clinical laboratory”^28^ were employed, using a modified long‐term replication study. IDEXX Laboratories does not provide specific control material for the Catalyst progesterone assay,^29^ and an analysis of in‐house produced control material was not possible due to the clinical setting, which depended on patient samples, and for financial reasons.

All concentrations described in the study design are from analysis with the DSI, and samples were divided into three groups according to concentration; high (>24 nmol/L), medium (12–24 nmol/L), and low (<12 nmol/L).^1^, ^4^, ^5^, ^8^, ^13^

#### 2.3.1 Linearity

For the linearity study, two samples with a high concentration (mean concentration of 49.4 and 33.3 nmol/L, analyzed on DSI, respectively) were used. A trained biomedical analyst diluted samples manually in a 20% step with left‐over refrigerated plasma taken from a healthy castrated male or female dog sampled for other reasons. All samples were diluted at the same time and analyzed in duplicate. Mean values were compared with calculated values, and recovery was calculated. For recovery upon addition, two samples with low progesterone concentrations (mean 0.6 and 1.1 nmol/L, analyzed on the DSI) and one sample with a high concentration (mean 38.9 nmoL/L, analyzed on the DSI) were used. Two hundred and fifty microliter aliquots of each low sample were used. For the study on addition using a high concentration sample, the volume needed to reach a progesterone concentration of at least 10 nmol/L was calculated and then added to the samples, which were analyzed in duplicate.^25^ The mean values of duplicates were compared with the calculated value, and recovery was calculated.

#### 2.3.2 Interference

A hemolysate of blood collected in a lithium‐heparin tube from another healthy male, mixed‐breed dog, was made using the osmotic shock procedure following guidelines in CSLI EP7‐A2.^27^ Blood was collected in a lithium‐heparin tube and centrifuged. The plasma was discarded and replaced with isotonic saline, and the tube was centrifuged again. The washing procedure was repeated twice, and the cells were diluted with distilled water and frozen overnight. The next day, the cell suspension was thawed, centrifuged, and the pellet was discarded. The hemoglobin concentration of the hemolysate, analyzed on the Sysmex xt2000i (Sysmex Nordic, Landskrona, Sweden), was 143 g/L.^27^ A patient sample with a mean progesterone concentration of 7.2 nmol/L was separated into two aliquots of 300 μL. Ten microliters of the hemolysate was added to one aliquot, and 10 μL of plasma from a healthy male was added to the other aliquot to account for possible dilution. The hemoglobin concentration of the sample with hemolysate was 8.0 g/L. The aliquots were analyzed in duplicate.

Two patient samples were separated into 300 μL aliquots and analyzed in duplicate before and after adding 10 μL of intra‐lipid (Fresenius Kabi; 200 mg/mL), or a corresponding amount of plasma from a healthy male mixed‐breed dog to account for possible dilution, as described above.

#### 2.3.3 Stability

The stability of lithium‐heparin plasma was investigated in two samples with low progesterone concentrations (mean 11.0 and 7.8 nmoL/L). Each sample was centrifuged directly after sampling and divided into two aliquots. One aliquot was analyzed within 30 minutes, after 12, and after 24 hours at room temperature. The other aliquot was frozen at −80°C directly after centrifugation and thawed and analyzed within 3 months after blood sampling. All samples were analyzed in duplicate, and a mean concentration and percentage of the original concentration were calculated.

#### 2.3.4 Precision

Precision was evaluated using four samples with low, medium, medium‐high, and high concentrations. Initially, the sample with a high concentration had a concentration above the reportable range for the DSI and was diluted 1:1 with plasma from a healthy, castrated female dog sampled for other reasons that day. From each sample, five aliquots were stored at −80°C. One aliquot each was thawed on 5 consecutive days and analyzed in five replicates over 2 hours. Each analysis took around 15 minutes. The standard deviation (SD), repeatability, and within‐laboratory imprecision were calculated. Results were tested for outliers by calculation of Grubb's lower and higher limits, and results that were outside these limits were excluded.^26^

#### 2.3.5 Method Comparison

A method comparison study was conducted with fresh samples from 26 dogs, collected on 39 occasions. On each occasion, serum was analyzed with CLIA and plasma with DSI. All analyses were performed in duplicate. Plasma samples were analyzed directly after centrifugation, and serum samples within 4 hours. Samples were kept at room temperature (18–25°C) until analysis, except samples used for mass spectrometry that were frozen at −80°C. For mass spectrometry, 19 of the samples with concentrations within the reportable range for the DSI were used. A validated protocol for the analysis of progesterone in dog plasma was executed. Briefly, samples were prepared by spiking with a corresponding deuterated internal standard followed by liquid‐liquid extraction with tert‐butyl methyl ether (MTBE) after protein precipitation with methanol prior to the analysis. During the extraction, the analyte was protected against oxidation by the addition of 0.05 mg/mL butylated hydroxytoluene (BHT) to MTBE, followed by derivatization with methoxyamine. The analysis was performed using an ultra‐performance supercritical fluid chromatography–tandem mass spectrometry (UPSFC–MS/MS, ACQUITY UPC^2^; Waters Corporation, Milford, MA, USA) system coupled with a Xevo TQ‐S triple quadrupole mass spectrometer (Waters, Milford, MA). Separation of progesterone was accomplished using the Acquity‐UPC^2^ BEH column (150 mm × 3.0 mm, 1.7 μm; Waters).^17^, ^30^

### 2.4 Statistical analysis

Statistical analysis was performed using Analyse‐it for Microsoft Excel Office 365 (Analyse‐it Software, Leeds, UK). For linearity testing, a visual evaluation of results and calculation of recovery were performed. For the evaluation of precision, a one‐way ANOVA was used according to EP‐15.^26^ For the method comparison among DSI, CLIA, and mass spectrometry, Passing‐Bablok regression analysis, and Bland‐Altman plot were used. The residuals were evaluated using the CUSUM test and were randomly and normally distributed. Bias was calculated after testing for normality using the Shapiro‐Wilks test. For stability and interference testing, too few samples were analyzed for meaningful statistical analysis.

## 3 RESULTS

A population of 29 healthy intact female dogs of 22 different breeds with a mean age of 4.7 years [+/− 2.3 years], were included. The following breeds were represented: Welsh Corgi Pembroke (5), Finnish Lapphund (1), English Springer Spaniel (1), Golden Retriever (1), Shetland Sheepdog (3), German Shepherd (1), Dachshund kaninchen, smooth‐haired (1), Welsh Corgi Cardigan (1), Gordon Setter (1), Rhodesian Ridgeback (2), American Bully (1), Miniature Schnauzer, pepper and salt (1), Border Terrier (1), Irish Water Spaniel (1), Schnauzer, pepper and salt (1), Irish Red Setter (1), Bichon Frisé (1), Border Collie (1), Flatcoated Retriever (1), Nova Scotia Duck Tolling Retriever (1), Bedlington Terrier (1), Malinois (1).

### 3.1 Linearity

**FIGURE 1:**
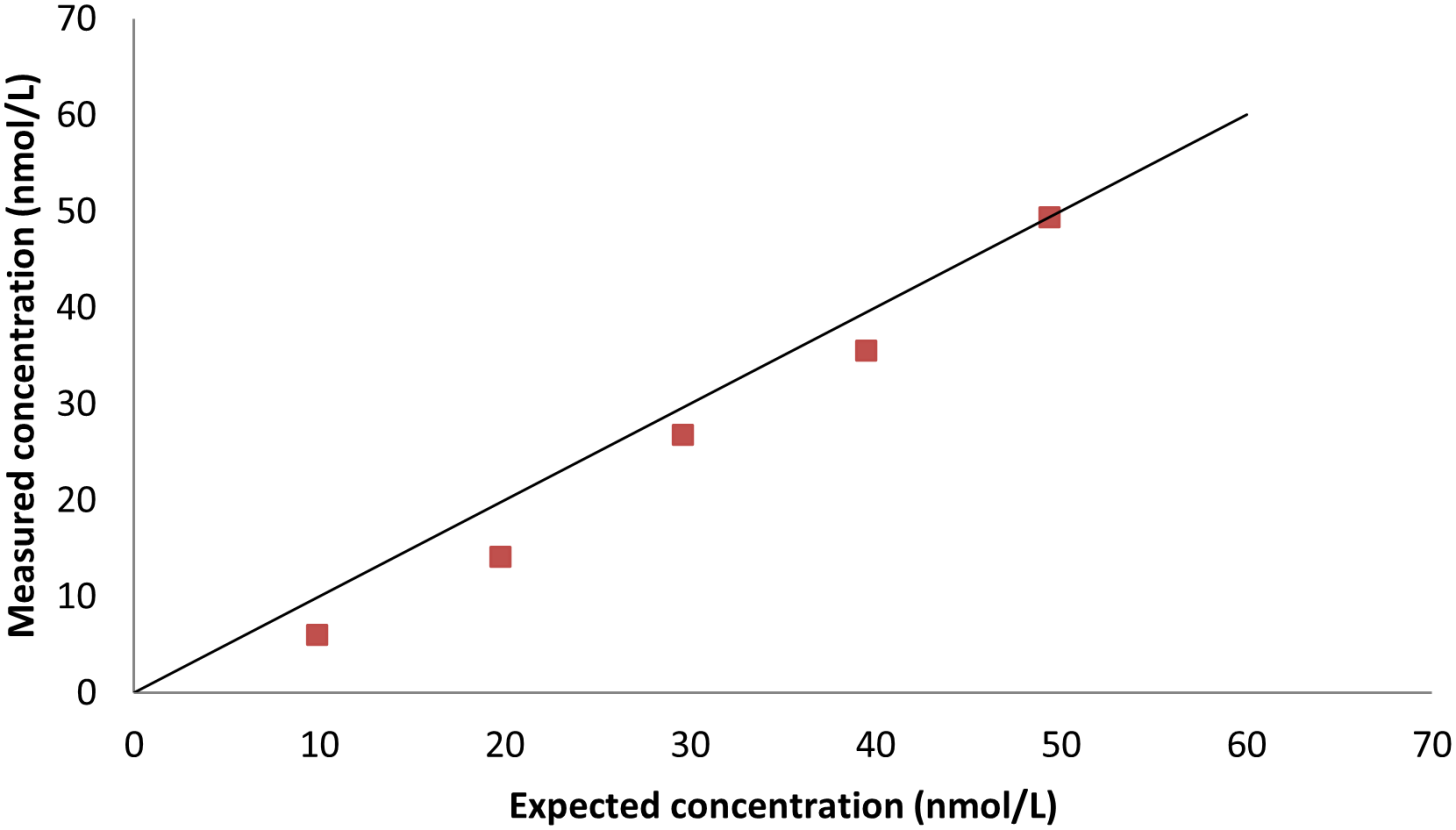
Linearity under dilution using the dry‐slide immunoassay (DSI) with a plasma sample with a mean progesterone concentration of 49.4 nmol/L.

**FIGURE 2:**
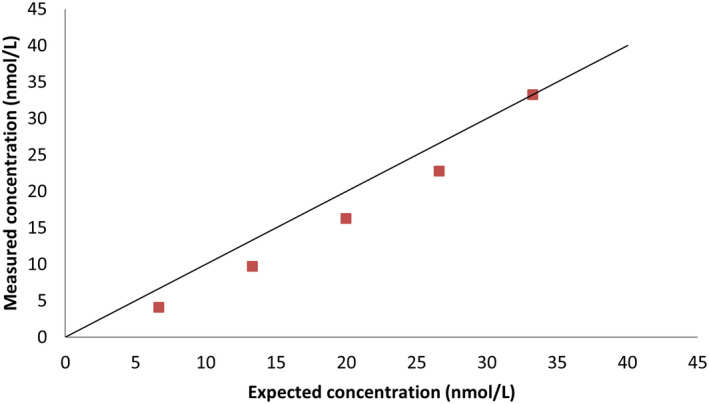
Linearity under dilution using the dry‐slide immunoassay (DSI) with a plasma sample with a mean progesterone concentration of 33.3 nmol/L.

For the sample with a concentration of 49.4 nmol/L, recovery under dilution was 83% (61%–100%,). For the sample with a concentration of 33.3 nmol/L, it was 80% (60%–100%). Results are shown in Figures 1 and 2. A sample with a concentration of 62.9 nmol/L was initially chosen for dilution, but one analytic result for this sample was above the reportable range on duplicate analyses in the dilution study and was, therefore, excluded.

For the study on addition, the sample with an initial concentration of 1.1 nmol/L had a recovery of 83%, and for the sample with an initial concentration of 0.6 nmol/L, the recovery was 76%.

### 3.2 Interference

In the interference study for hemolysis, the progesterone concentration was measured to 6.1 nmol/L after the addition of the hemolysate in the sample, with an initial mean progesterone concentration of 7.2 nmol/L.

In the interference study for lipemia, the mean differences of 2.6 nmol/L (an increase from 16.15 to 18.75) and 0.45 nmol/L (a decrease from 5.8 to 5.4) were seen in the two samples. The resulting concentrations ranged between 92 and 116% of the initial concentrations.

### 3.3 Stability

In the stability study, the progesterone concentration varied between 57 and 96% of the original concentration, as shown in Table 1.

**TABLE 1:** Results from the stability study. The plasma samples analyzed within 30 minutes and after 12 and 24 hours were kept at room temperature. The plasma samples analyzed after 3 months were kept at −80°C

	Concentration 1 (nmol/L)	Concentration 2 (nmol/L)	Mean	% of initial concentration
P1				
Within 30 minutes	7.8	7.7	7.75	NA
After 12 hours	6.7	8.2	7.45	96
After 24 hours	6.9	6.8	6.85	88
After 3 months	5.6	5.7	5.65	73
P2				
Within 30 minutes	10.3	11.7	11.0	NA
After 12 hours	6.1	6.4	6.25	57
After 24 hours	7.7	^a^	7.7	70
After 3 months	10.3	8.9	9.1	83

^a^ For one sample from patient P2, an analytic error occurred in one of the duplicate analyses for the sample after 24 hours, resulting in the loss of sample material.

### 3.4 Precision

The results from the precision study are shown in Table 2. For the sample with the low concentration, one outlier was identified according to EP15^15^ and excluded. For the four concentration levels, repeatability ranged between 8.0% and 10.8%, and within‐laboratory imprecision was 8.8%–11.1%.

**TABLE 2:** Repeatability and within‐laboratory imprecision for four concentration levels

Precision	Repeatability			Within‐laboratory variation		
Sample	Mean (nmol/L)	SD (nmol/L)	CV (%)	Mean (nmol/L)	SD (nmol/L)	CV%
Low^a^	6.1	0.64	10.5	6.1	0.64	10.5
Medium	19.2	2.07	10.8	19.2	2.07	10.8
Medium‐high	32.9	2.90	8.8	32.9	3.65	11.1
High	48.1	3.85	8.00	48.1	4.19	8.8

^a^ For the low plasma sample, one outlier was identified and excluded according to EP‐15.^26^

A sample with a mean concentration of 53.7 nmol/L was initially chosen for the precision study, but 11/25 analyses yielded a result above the instrument's reportable range, as shown in Table 3. Therefore, another sample with a mean concentration above the reportable range was diluted, as stated earlier.

**TABLE 3:** Results from precision study of a plasma sample with high progesterone concentration. Forty‐four percent of the results were above the reportable range for the DSI

Day	1	2	3	4	5
rep1	>63.6	>63.6	>63.6	55.8	61.2
rep2	62	63	58.8	>63.3	>63.6
rep3	59.4	63.4	60.5	>63.6	>63.6
rep4	60	>63.6	59.2	>63.6	61.8
rep5	58	>63.6	57.3	59.4	>63.6

Abbreviations: DSI, dry‐slide immunoassay; rep, replicate.

### 3.5 Method comparison

**FIGURE 3:**
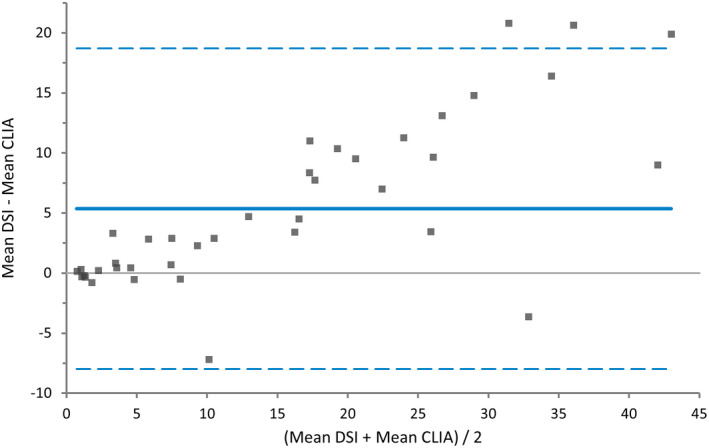
Bland‐Altman difference plot for the progesterone analysis in canine plasma and serum with the dry‐slide immunoassay (DSI) and chemiluminescence assay (CLIA) (n = 39). The mean bias was 5.4, the 95% lower limit of agreement (LOA) was −7.99 [−11.8 to −4.18], and the 95% upper LOA was 18.72 [14.92 to 22.53].

Results from the method comparison study between DSI and CLIA are shown graphically in the Bland‐Altman difference plot in Figure 3, where a significant proportional error is seen. Using the Passing‐Bablok fit, the correlation coefficient was 0.94. Evaluation of the 95% confidence intervals for both the intercept and slope revealed constant and proportional error, respectively.

Results for the mass spectrometry analysis were compared with CLIA and DSI separately, and the Passing Bablok fit and Bland‐Altman are presented for each below.

**FIGURE 4:**
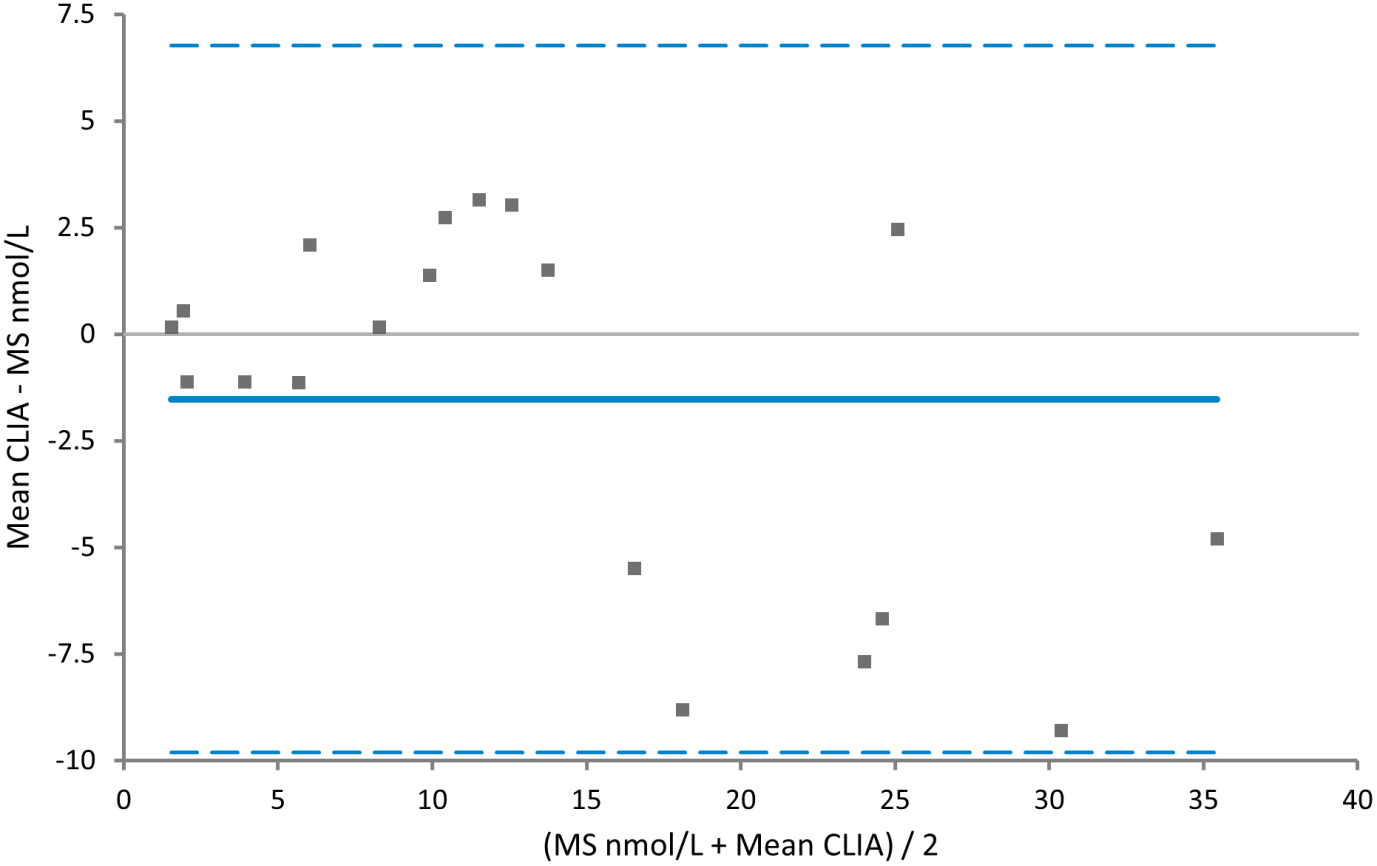
Bland‐Altman difference plot for progesterone analysis in canine serum and plasma measured with chemiluminescence assay (CLIA) and mass spectrometry (n = 19). The mean bias was −1.5, the 95% lower limit of agreement (LOA) was −9.11 [−13.36 to‐6.26], and the 95% upper LOA was 6.77 [3.22 to 10.31].

A correlation coefficient between CLIA and mass spectrometry of 0.94 was seen. The evaluation of the 95% confidence intervals for the intercept and slope showed no constant or proportional error. In the Bland‐Altman difference plot, the CLIA tended to underestimate progesterone concentrations at values above 15–20 nmol/L (Figure 4).

**FIGURE 5:**
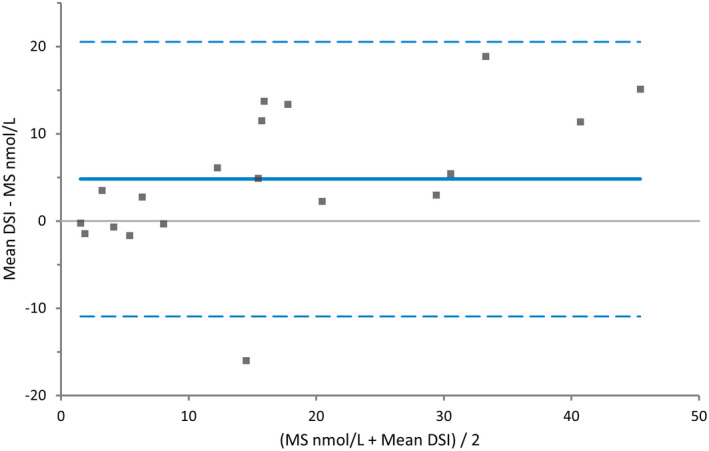
Bland‐Altman difference plot for progesterone analysis in canine plasma using the dry‐slide immunoassay (DSI) and mass spectrometry (n = 19). The mean bias was 4.8, the 95% lower limit of agreement (LOA) was −10.92 [−17.65 to −4.19], and the 95% upper LOA was 13.81 to 27.27].

In the comparison between DSI and mass spectrometry, a correlation coefficient of 0.88 was seen, and a proportional error was found. For the Bland‐Altman plot, the DSI had a tendency to overestimate concentration ranges above 12–15 nmol/L (Figure 5).

## 4 DISCUSSION

Results from this clinical validation study examining the analysis of progesterone using IDEXX Catalyst dry‐slide immunoassay showed that the method is linear up to an approximate concentration of 50 nmol/L. In the method comparison study, there was a proportional bias when comparing DSI with CLIA and mass spectrometry, and the DSI did not correlate as well with mass spectrometry as the CLIA did. Both repeatability and within‐laboratory variation were acceptable in all measured ranges.

The reportable range of the assay is set to 0.6–63.6 nmol/L according to the manufacturer's information.^18^ It is noteworthy that a sample with a mean concentration of 53.7 nmol/L on duplicate analyses using DSI yielded results above the upper limit of the reportable range in 44% of the analyses when used in the precision study. With the relatively low imprecision of 8%–8.8% for the sample with a high mean concentration (48.1 nmol/L), and a repeatability of 8.0%–10.8%, and within‐laboratory imprecision of 8.8%–11.1% for all concentration ranges, the results above the reportable range for 44% of analyses of the sample with a mean concentration of 53.7 nmol/L, could not be explained by imprecision only. Furthermore, the sample with an initial concentration of 62.9 nmol/L also produced results above the reportable range on duplicate analyses in the dilution study. This finding raises concerns about the method's linearity in the upper reportable range. Since the study was dependent on patient samples, another sample with a concentration of around 60 nmol/L could not be obtained. Thus, we could not assess linearity in the entire reportable range. Linearity in the lower range was deemed excellent. Since medical decisions are made using values in the middle of the reportable range, that is, with progesterone values around or above 12–24, and progesterone values in the lowest range are not used clinically, a detection limit study was not conducted.^1^, ^7^, ^8^, ^10^, ^11^, ^13^

Recovery after spiking was not possible since a standardized progesterone solution was not available, and a study using the addition of a sample with a high progesterone concentration was performed instead. The recovery was 76% and 83%, which is acceptable but indicates that there was a potential matrix effect to consider.^25^ The sample with the high progesterone concentration that was used for the addition study was initially analyzed with the DSI since the validation study was based on the use of plasma. Therefore, samples could not be analyzed with CLIA concurrently, since the use of plasma for progesterone analysis with CLIA was not validated. The inborn imprecision of the assay might affect the measured concentration, thereby affecting the calculated recovery after addition.

Interference was evaluated for hemolysis and lipemia, with results indicating no significant effect of hemolysis or lipemia. The manufacturer proposed a possible effect of severe hemolysis but claimed that the analysis is robust in cases of lipemia, which agrees well with the findings in the present study.^31^ A limitation was that there were too few samples for statistical analysis of the interference study. A larger sample size would be needed to make conclusions regarding the possible effect of hemolysis. Furthermore, lipemia was induced with intra‐lipid, which would be a non‐representative measure of naturally occurring lipemia since the lipid emulsion manufactured on soy oil could have a different effect on the assay than triglycerides produced in‐vivo.

In the investigation of stability, there was a tendency toward a decrease in plasma progesterone concentrations, in contrast to results in previous studies where a statistical decrease was noted.^23^, ^24^ This could be due to a difference in assays or storage conditions. It would be interesting to evaluate stability with a larger sample size to estimate how long a sample can be stored at room temperature before analysis without a result being affected. However, the clinical relevance of progesterone stability in plasma samples is limited since progesterone analysis with plasma using DSI is most often used when quick results are required, and the short turn‐around time is one of the largest advantages of this analysis.

From the precision study, a repeatability of 8.0%–10.8% and within‐laboratory imprecision ranged between 8.8% and 11.1% for low, medium, medium‐high, and high concentrations, which was deemed acceptable for use in clinical practice, and in line with the manufacturer's claim and the study by Zuercher et al.^18^, ^22^ The results from the precision study also agree with those described for other available in‐house assays.^14^, ^16^, ^20^ The high sample with an initial mean concentration of 53.7 nmol/L using the DSI could not be used since 11/25 of analyses rendered results above the measurement range (>63.6 nmol/L). Considering that results in the lower concentration ranges gave a repeatability of 8.0%–10.8%, the sample with a concentration of 53.7 nmol/L should have yielded results within the reportable range of the DSI if the repeatability was the same across the reportable range. Since the study was dependent on patient samples from patients that were most often presented for ovulation timing to assist with natural breeding, it was not possible during the time frame of the study to collect samples with a concentration between 32.9 and 53.7 nmol/L for the precision study. A sample with a concentration above the reportable range was diluted before repeatability, and within‐laboratory variation could be calculated. The repeatability and within laboratory variation of that sample were found to be similar to that reported for the other concentration ranges and a previously published study.^22^ Although, it is uncertain if the dilution of the sample could influence precision, due to potential matrix effects. Another study with undiluted patient samples with a concentration around 50 nmol/L would be interesting.

The method comparison study with DSI and CLIA revealed a mean bias of 5.4 with a proportional error, shown visually in Figure 3. The correlation coefficient of 0.94, together with the proportional error indicates that direct comparison between the two instruments is not recommended, and a conversion factor cannot be used. This finding is in line with the previous method comparison between the DSI and CLIA where a proportional error was also seen, and the DSI measured higher progesterone concentrations earlier when two bitches were followed during heat.^22^ The results from both our study and the previous study on the DSI are like the results shown by Gloria et al where the ELISA test yielded higher results than the CLIA.^15^ There are several reasons for the differences in the measured concentrations. First, serum was used for analysis with CLIA, and lithium‐heparin and plasma were used for analysis with DSI; however, the sample material used was the same as in the study published by the manufacturer. In the study by Zuercher et al, serum was used for analysis with both methods, and proportional error was still seen.^18^, ^22^ Lithium‐heparin plasma was chosen for analyses with DSI in this study, as this is a common sample material that generates rapid results and is recommended by the manufacturer.^18^ In a previous study, the difference in progesterone concentrations between heparinized plasma and serum samples was not detected, whereas other studies found a difference.^23^, ^24^, ^32^ Although, a comparison of those studies is problematic since samples were handled under different conditions and analyzed with different assays.^23^, ^24^, ^32^

A direct comparison between CLIA and DSI was not performed in the manufacturer's study, which makes a comparison of their results for the CLIA and DSI difficult.

The method comparison with DSI and mass spectrometry showed a mean bias of 4.8 with proportional error, and the DSI overestimated values above 15–18 nmol/L (Figure 4). A correlation coefficient of 0.88 indicates that these two methods are not comparable. The correlation coefficient was also significantly lower than that described in the study published by the manufacturer, where a correlation coefficient of 0.98 and 0.99 was seen between mass spectrometry and both DSI and CLIA.^18^

Interestingly CLIA underestimated values in the same concentration ranges, and the bias was −1.5 between CLIA and mass spectrometry, also with proportional error (Figure 5). This underestimation could explain the difference and part of the magnitude of the proportional bias seen when comparing CLIA and DSI.

Mass spectrometry was chosen as the gold standard in this study, but even this method can have issues with matrix effects, both with lower and higher concentrations measured, which should be considered when evaluating results of a method comparison study.^33^

The proportional error seen in the method comparison study between the DSI and both mass spectrometry and CLIA could indicate that the DSI is more sensitive to matrix effects than the other two methods, and it is possible that this would affect the results due to interference from substances in plasma.^34^

Heterophilic antibodies have previously been shown to interfere with immunoassays in both human and veterinary medicine.^34^, ^35^ Interference has been shown for both dogs and cats.^36^ Recently interference using an assay for the measurement of anti‐Müllerian hormone has been identified.^37^ Interference with immunoassays used for progesterone analysis has not been investigated in dogs but has been seen in human assays.^38^ Interference from heterophilic antibodies in the DSI is therefore possible, and future studies on the impact of heterophilic antibodies on progesterone analysis in canine blood are warranted.

The reported repeatability and within‐laboratory imprecision, in conjunction with the results from the linearity study, indicate that the DSI can be used to determine ovulation since ovulation typically occurs at concentrations of below 25 nmol/L, and both the precision and linearity in that concentration range are deemed acceptable.^4^, ^13^ Determining the time for artificial insemination (AI) with frozen semen could be problematic, as AI typically is performed after ovulation, at progesterone concentrations about 30–80 nmol/L, which is close to, or above, the upper limit of the reportable range for the DSI.^10^, ^13^, ^18^ There is an indication for larger imprecision at the highest concentration range. Therefore, clinical decisions regarding the timing of AI are not recommended for progesterone concentrations higher than those at ovulation, since an overestimation of the progesterone concentration could lead to AI being performed untimely.^7^ Furthermore, linearity in the higher reportable range could not be assessed, and that should be evaluated before using the DSI for AI based on higher progesterone concentrations can be recommended.

In clinical use, when breeders visit different clinics, comparison between the CLIA and DSI may be possible at the lower concentration ranges, with careful consideration in interpreting results from different methods and with different sample materials. Separate interpretation guidelines would need to be established for higher ranges.

Since the study was based on patient samples, it was difficult to collect samples representing the whole measurement range, and the samples were collected and analyzed over a longer period, which could affect the results generated in the method comparison study with CLIA and mass spectrometry. Furthermore, only 39 samples were used for the method comparison; a larger sample population could generate stronger statistical results. As mentioned earlier, this is also true for the stability, recovery, and interference studies. In future studies with more samples at relevant concentration levels, it would be interesting to make conclusions on both interference and stability.

## 5 CONCLUSIONS

Progesterone analysis with the IDEXX Catalyst DSI was validated with acceptable accuracy and precision, although there was a concern for a possible matrix effect. The error between DSI and CLIA was proportional and too large to allow for the comparison of results. The DSI overestimated results from approximately 15–20 nmol/L when compared with mass spectrometry, whereas CLIA tended to underestimate the results. Interference studies of hemolysis and lipemia did not have statistical power to make conclusions, but a significant effect was not seen. Stability was evaluated in two samples, and a small decrease was seen over time, which clinicians should bear in mind when using this method. Until further studies have been carried out, samples should be analyzed without delay.

Results from this study indicate that the IDEXX Catalyst progesterone assay can be used to assess the time of ovulation, but it can be problematic for assessing the optimal time for AI with frozen semen based on higher progesterone concentrations. The linearity and precision studies performed indicated that the assay's linearity and reportable range do not cover the progesterone concentrations used for the timing of AI, and that the imprecision of the upper measurement range is too large.

## DISCLOSURE

The article was funded by AniCura Research fund.

## References

[1] Concannon PW . Reproductive cycles of the domestic bitch. Anim Reprod Sci. 2011;124(3–4):200‐210. 21055888 10.1016/j.anireprosci.2010.08.028

[2] Kutzler MA , Mohammed HO , Lamb SV , Meyers‐Wallen VN . Accuracy of canine parturition date prediction from the initial rise in preovulatory progesterone concentration. Theriogenology. 2003;60(6):1187‐1196. 12935856 10.1016/s0093-691x(03)00109-2

[3] Concannon PW , Hansel W , Visek WJ . The ovarian cycle of the bitch: plasma estrogen, LH and Progesterone. Biol Reprod. 1975;13(1):112‐121. 1222178 10.1095/biolreprod13.1.112

[4] Groppetti D , Aralla M , Bronzo V , Bosi G , Pecile A , Arrighi S . Periovulatory time in the bitch: what's new to know? Comparison between ovarian histology and clinical features. Anim Reprod Sci. 2015;152:108‐116. 25510561 10.1016/j.anireprosci.2014.11.008

[5] Hase M , Hori T , Kawakami E , Tsutsui T . Plasma LH and progesterone levels before and after ovulation and observation of ovarian follicles by ultrasonographic diagnosis system in dogs. J Vet Med Sci. 2000;62(3):243‐248. 10770594 10.1292/jvms.62.243

[6] Fortune JE , Willis EL , Bridges PJ , Yang CS . The periovulatory period in cattle: progesterone, prostaglandins, oxytocin and ADAMTS proteases. Animal Reprod. 2009;6(1):60‐71. PMC2853051 20390049

[7] Hahn SE , Jo YK , Jin YK , Jang G . Timing of fertile period for successful pregnancy in American bully dogs. Theriogenology. 2017;104:49‐54. 28818583 10.1016/j.theriogenology.2017.07.034

[8] Goodman M . Ovulation timing. Concepts and controversies. Vet Clin North Am Small Anim Pract. 2001;31(2):219‐235. 11265488 10.1016/s0195-5616(01)50201-6

[9] Steckler D , Nothling JO , Harper C . Prediction of the optimal time for insemination using frozen‐thawed semen in a multi‐sire insemination trial in bitches. Anim Reprod Sci. 2013;142(3–4):191‐197. 24128644 10.1016/j.anireprosci.2013.09.013

[10] Linde‐Forsberg C . Accurate monitoring of the Oestrus cycle of the bitch for artificial insemination. Proceedings of the WSAVA XIX world congress Durban. 1994;601‐604.

[11] Root Kustritz MV . Managing the reproductive cycle in the bitch. Vet Clin North Am Small Anim Pract. 2012;42(3):423‐437. 22482809 10.1016/j.cvsm.2012.01.012

[12] Moxon R , Copley D , England GC . Quality assurance of canine vaginal cytology: a preliminary study. Theriogenology. 2010;74(3):479‐485. 20494431 10.1016/j.theriogenology.2010.02.031

[13] Hollinshead F , Hanlon D . Normal progesterone profiles during estrus in the bitch: a prospective analysis of 1420 estrous cycles. Theriogenology. 2019;125:37‐42. 30388469 10.1016/j.theriogenology.2018.10.018

[14] Renton JP , Boyd JS , Harvey MJ , Ferguson JM , Nickson DA , Eckersall PD . Comparison of endocrine changes and ultrasound as means of identifying ovulation in the bitch. Res Vet Sci. 1992;53(1):74‐79. 1410822 10.1016/0034-5288(92)90088-j

[15] Gloria A , Contri A , Carluccio A , Robbe D . Blood periovulatory progesterone quantification using different techniques in the dog. Anim Reprod Sci. 2018;192:179‐184. 29545003 10.1016/j.anireprosci.2018.03.006

[16] Chapwanya A , Clegg T , Stanley P , Vaughan L . Comparison of the Immulite and RIA assay methods for measuring peripheral blood progesterone levels in greyhound bitches. Theriogenology. 2008;70(5):795‐799. 18579195 10.1016/j.theriogenology.2008.05.047

[17] Holst BS , Kushnir MM , Bergquist J . Liquid chromatography‐tandem mass spectrometry (LC‐MS/MS) for analysis of endogenous steroids in the luteal phase and early pregnancy in dogs: a pilot study. Vet Clin Pathol. 2015;44(4):552‐558. 26595760 10.1111/vcp.12301

[18] Bilbrough G & Glavan, T , eds. Idexx: Catalyst Progesterone for in‐House Measurement of Progesterone in Plasma from bitches. 2019. https://www.idexx.com/files/catalyst‐progesterone‐white‐paper‐en.pdf. Accessed March 27, 2019.

[19] Stanczyk FZ , Clarke NJ . Advantages and challenges of mass spectrometry assays for steroid hormones. J Steroid Biochem Mol Biol. 2010;121(3–5):491‐495. 20470886 10.1016/j.jsbmb.2010.05.001

[20] Brugger N , Otzdorff C , Walter B , Hoffmann B , Braun J . Quantitative determination of progesterone (P4) in canine blood serum using an enzyme‐linked fluorescence assay. Reprod Domest Anim. 2011;46(5):870‐873. 21323757 10.1111/j.1439-0531.2011.01757.x

[21] Tripp KM , Verstegen JP , Deutsch CJ , et al. Validation of a serum immunoassay to measure progesterone and diagnose pregnancy in the west Indian manatee (*Trichechus manatus*). Theriogenology. 2008;70(7):1030‐1040. 18760463 10.1016/j.theriogenology.2008.06.024

[22] Zuercher J , Boes KM , Balogh O , Helms AB , Cecere JT . Comparison of a point‐of‐care analyzer with a chemiluminescent immunoassay for serum progesterone measurement in breeding management of the bitch. Front Vet Sci. 2021;8:660923. 34055950 10.3389/fvets.2021.660923 PMC8155301

[23] Volkmann DH . The effects of storage time and temperature and anticoagulant on laboratory measurements of canine blood progesterone concentrations. Theriogenology. 2006;66(6–7):1583‐1586. 16480764 10.1016/j.theriogenology.2006.01.024

[24] Tahir MZ , Thoumire S , Raffaelli M , Grimard B , Reynaud K , Chastant‐Maillard S . Effect of blood handling conditions on progesterone assay results obtained by chemiluminescence in the bitch. Domest Anim Endocrinol. 2013;45(3):141‐144. 23988180 10.1016/j.domaniend.2013.07.002

[25] Flatland B , Freeman KP , Friedrichs KR , et al. ASVCP quality assurance guidelines: control of general analytical factors in veterinary laboratories. Vet Clin Pathol. 2010;39(3):264‐277. 21054473 10.1111/j.1939-165X.2010.00251.x

[26] Wayne PA . User verification of precision and estimation of bias; approved guideline – third edition. CLSI Document EP15‐A3. 2014;34(12).

[27] Wayne PA . Interference testing in clinical chemistry; approved guideline ‐ second edition. CSLI Document EP7‐A2. 2005;25(27).

[28] Jensen AL , Kjelgaard‐Hansen M . Method comparison in the clinical laboratory. Vet Clin Pathol. 2006;35(3):276‐286. 16967409 10.1111/j.1939-165x.2006.tb00131.x

[29] Idexx . Catalyst progesterone quick reference guide. 2019. https://www.idexx.com/files/catalyst‐progesterone‐quick‐reference‐guide‐en.pdf. Accessed March 28, 2019.

[30] de Kock N , Acharya SR , Ubhayasekera S , Bergquist J . A novel targeted analysis of peripheral steroids by ultra‐performance supercritical fluid chromatography hyphenated to tandem mass spectrometry. Sci Rep. 2018;8(1):16993. 30451874 10.1038/s41598-018-35007-0 PMC6242962

[31] Idexx . Catalyst Dx Chemistry Analyzer Operator's Guide. https://www.idexx.com/files/catalyst‐dx‐operators‐guide‐en.pdf. Accessed May 15, 2020.

[32] Thuroczy J , Wolfling A , Tibold A , Balogh L , Janoki GA , Solti L . Effect of anticoagulants and sampling time on results of progesterone determination in canine blood samples. Reprod Domest Anim. 2003;38(5):386‐389. 12950690 10.1046/j.1439-0531.2003.00450.x

[33] Zhou W , Yang S , Wang PG . Matrix effects and application of matrix effect factor. Bioanalysis. 2017;9(23):1839‐1844. 29171768 10.4155/bio-2017-0214

[34] Selby C . Interference in immunoassay. Ann Clin Biochem. 1999;36(Pt 6):704‐721. 10586307 10.1177/000456329903600603

[35] Solter PF , Oyama MA , Sisson DD . Canine heterophilic antibodies as a source of false‐positive B‐type natriuretic peptide sandwich ELISA results. Vet Clin Pathol. 2008;37(1):86‐95. 18366550 10.1111/j.1939-165X.2008.00002.x

[36] Bergman D , Larsson A , Hansson‐Hamlin H , Svensson A , Holst BS . Prevalence of interfering antibodies in dogs and cats evaluated using a species‐independent assay. Vet Clin Pathol. 2018;47(2):205‐212. 29902338 10.1111/vcp.12612

[37] Bergman D , Larsson A , Hansson‐Hamlin H , Strom HB . Investigation of interference from canine anti‐mouse antibodies in hormone immunoassays. Vet Clin Pathol. 2019;48(Suppl 1):59‐69. 31318069 10.1111/vcp.12764

[38] Langlois F , Moramarco J , He G , Carr BR . Falsely elevated steroid hormones in a postmenopausal woman due to laboratory interference. J Endocr Soc. 2017;1(8):1062‐1066. 29264558 10.1210/js.2017-00191 PMC5686690

